# Supplementary material for: Taxonomic and functional turnover are decoupled in European peat bogs
Source: Nat Commun. 2017 Oct 27;8:1161. doi: 10.1038/s41467-017-01350-5 (PMC5660083; doi:10.1038/s41467-017-01350-5)
Supplement: Supplementary file 1 — Supplementary Information [file 41467_2017_1350_MOESM1_ESM.pdf]

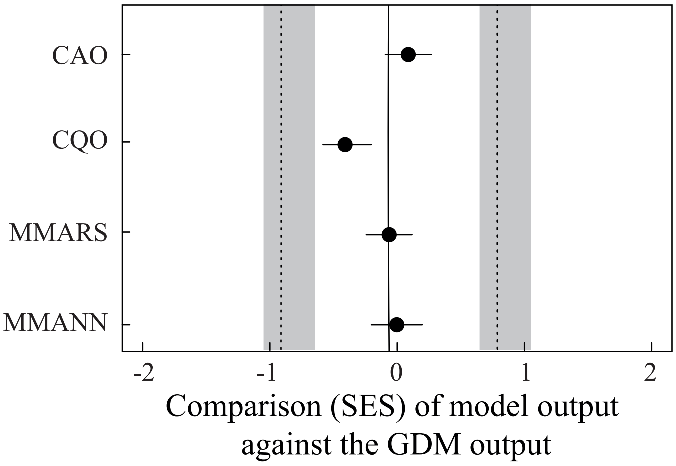

Supplementary Figure 1. Alternative model comparison. Model comparisons, comparing the results of general dissimilarity modelling (GDM) with four alternative community-level models. CAO, constrained additive ordination; CQO, constrained quadratic ordination; MMARS, multiresponse multivariate adaptive regression splines; MMAN, multiresponse multivariate artificial neural networks. For each model, the importance of the same ten environmental variables (latitude, longitude, MAT, MAP, TS, P:T_{warm}, PS, NO_y, NH_x and SO_x) in driving plant species turnover was extracted and compared against the outputs from GDM using paired analysis and standardized effect sizes (SES). Dashed lines and grey bars represent the mean level (+/- confident interval) where model outcomes would be different from those of GDM.

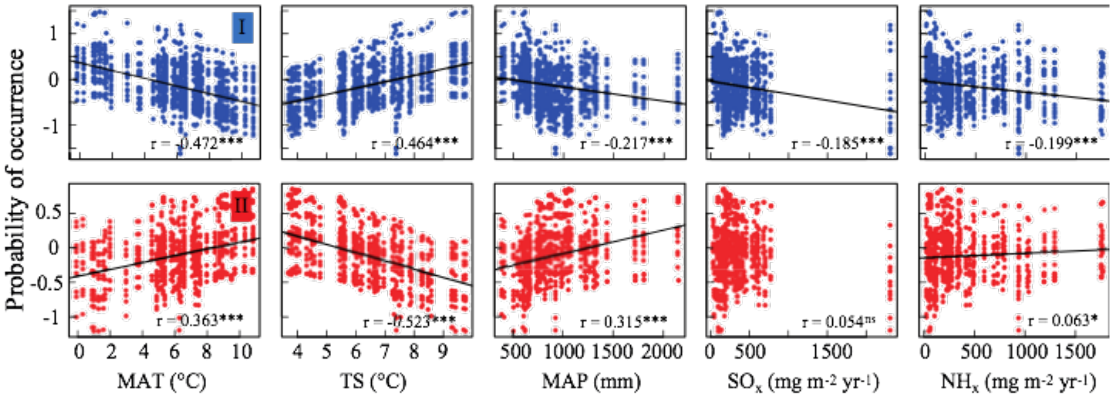

Supplementary Figure 2. Cluster specific plant responses to environmental variables. Relationships between the probability of peatland species occurrence and bioclimatic and atmospheric deposition variables for species in the two species co-response clusters (see main text Fig. 3; blue = cluster I, red = cluster II). Lines represent significant ($P \leq 0.05$) linear regressions.

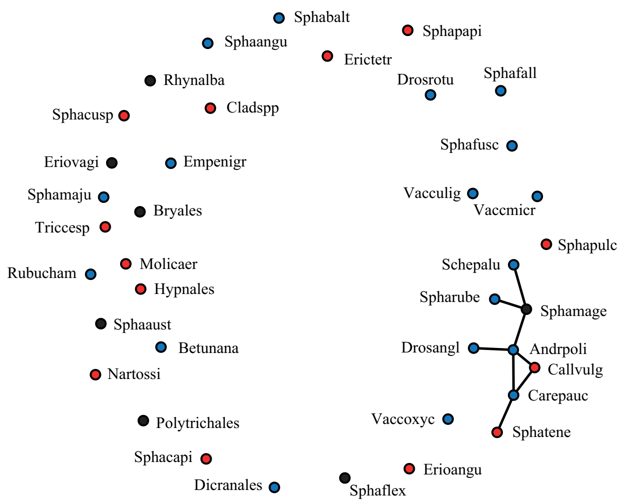

Supplementary Figure 3. Residual correlation cluster. Joint distribution modelling (JSDM) correlation cluster after environmental effects. Black lines indicate correlations between species with a correlation coefficient ≥ 0.6.

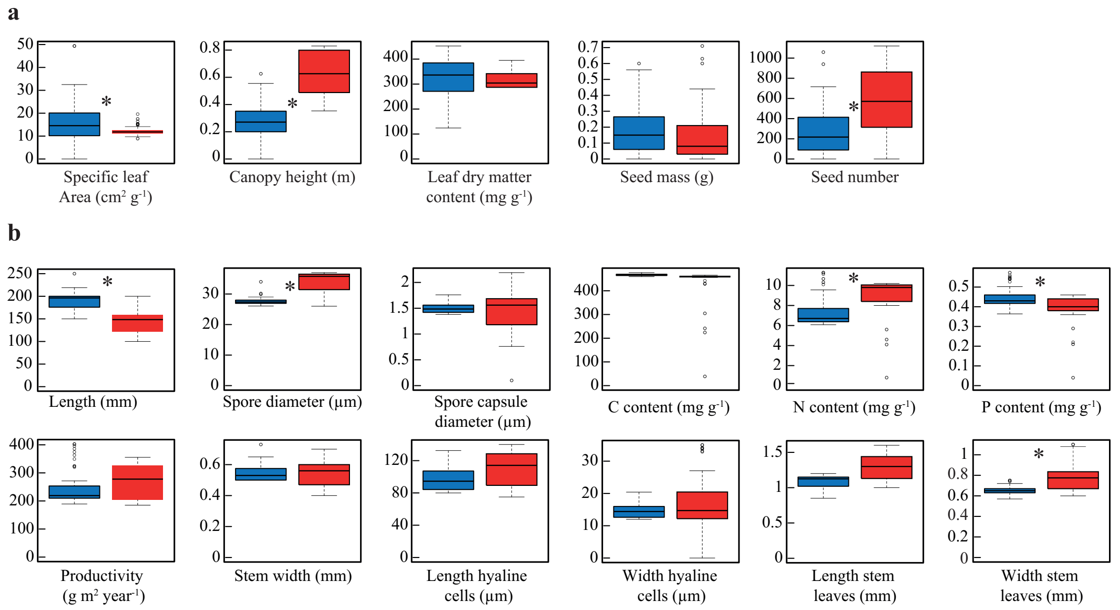

Supplementary Figure 4. Boxplot representation of plant functional trait values. Functional trait variations among the two co-response clusters for vascular plants (**a**) and *Sphagnum* mosses (**b**). Boxplots represent median, 25-75% quantiles (box), 95% quantiles (whiskers) and outliers (circles). SLA, specific leaf area; LDMC, leaf dry matter content. Two-sided ANOVA tests were used to assess significant differences among clusters (* P < 0.01). Cluster I: blue, Cluster II: red. Only quantitative traits are shown for vascular plants.

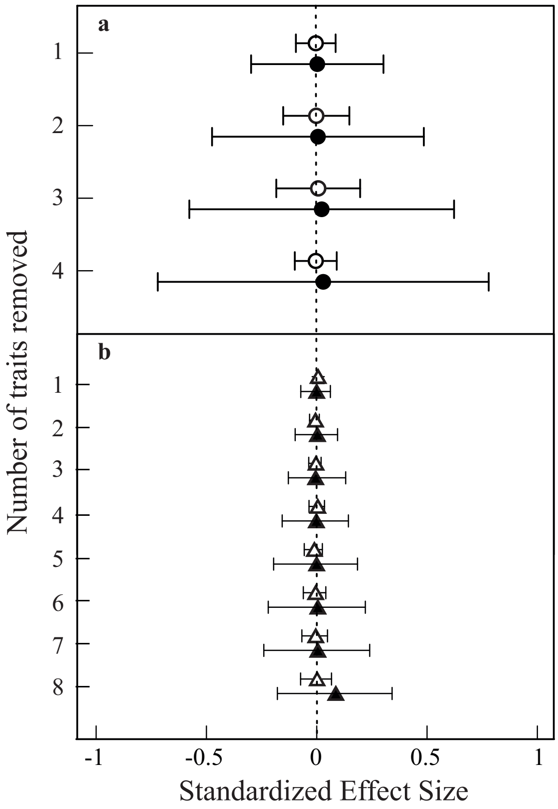

Supplementary Figure 5. Plant functional trait removal models. Comparison of functional redundancy (open symbols) and functional diversity (filled symbols) after randomly removing up to four vascular plants (**a**) or eight *Sphagnum* (**b**) plant traits (always leaving at least four traits in the dataset on which both indices are based). The values (± SEM) of both functional indices of the reduced trait dataset were tested against the values of the full dataset using paired analysis and standardized effect sizes (SES).

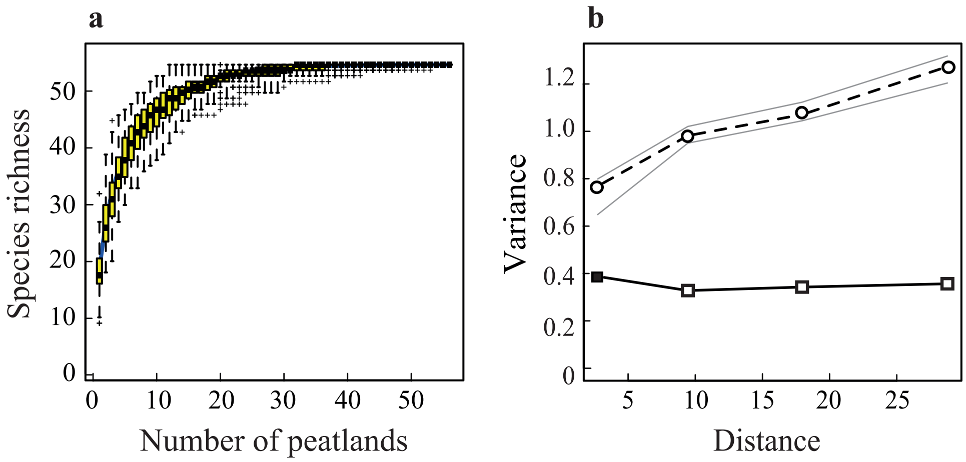

Supplementary Figure 6. Species-area curve and spatial autocorrelation. **a** Sample-based rarefaction curves of the number of plant species. **b** Sample based analysis on spatial autocorrelation between peatland sites based on their environmental dissimilarities. Only very close sites were spatially auto–correlated (filled symbols indicate significant autocorrelation). Dashed line indicates total variance. The grey line indicates its 95% confidence intervals. The solid line indicates the residual variance

Supplementary Table 1. Environmental gradient variables. Geographic, bioclimatic and atmospheric deposition variables used to describe environmental difference between peatland sites. Significant variables from stepwise (multiple linear) regressions of European peatland plant species richness, including geographical bioclimatic and total atmospheric deposition variables. Numbers indicate t-values. $^{(*)}P \leq 0.1$, $^{*}P \leq 0.05$, $^{**}P \leq 0.01$,$^{***}P \leq 0.001$.

		Range	S	$H_{Simpson}$
**Geographic**				
Latitude	Lat	46°20'N – 68°21'N	4.66^{***}	2.76^{**}
Longitude	Long	07°37'W – 30°56'E	2.15^{*}	1.39
Altitude (m a.s.l)	Alt	-2 – 1879 m. a.s.l.	—	2.19^{*}
**Bioclimatic**				
Mean annual temperature (°C)	MAT	-0.1 – 10.8 °C	2.10^{*}	$1.96^{(*)}$
Seasonality in temperature	TS	3.7 – 9.8 °C	—	—
Mean annual precipitation (mm)	MAP	391 – 2147 mm	—	—
Seasonality in precipitation	PS	12 – 44 %	–1.83	$–3.05^{**}$
Lang's moisture index; $P{:}T_{warm}$	$P{:}T_{warm}$	11.3 – 40.9 mm $°C^{-1}$	4.37^{***}	—
**Atmospheric deposition**				
Total sulphur deposition	SO_x – tot	40 – 2306 mg m^{-2} yr^{-1}	—	1.58
Total oxidized nitrogen deposition	NO_y – tot	39 – 781 mg m^{-2} yr^{-1}	—	$–1.69^{(*)}$
Total reduced nitrogen deposition	NH_x – tot	22 – 1770 mg m^{-2}	—	—
r^2_{adj}			0.38	0.17

Supplementary Table 2. List of cluster associated European peat bog plants. Species associated with the co-response clusters in the joint species distribution modelling (JSDM)

**Cluster I**	**Cluster II**	**Non-cluster**
Vascular plants		
Andromeda polifolia	*Calluna vulgaris*	*Eriophorum vaginatum*
Betula nana	*Eriophorum angustifolium*	*Rhynchospora alba*
Carex pauciflora	*Erica tetralix*†	
Drosera anglica	*Molinia caerulea*	
Drosera rotundifolia	*Narthecium ossifragum*	
*Empetrum nigrum**	*Trichophorum cespitosum*	
Rubus chamaemorus		
Scheuchzeria palustris		
Vaccinium oxycoccus		
Vaccinium uliginosum		
Vaccinium microcarpon		
Sphagnum mosses		
Sphagnum angustifolium	*Sphagnum capillifolium*	*Sphagnum austinii*
Sphagnum balticum	*Sphagnum cuspidatum*	*Sphagnum flexuosum*
Sphagnum fallax	*Sphagnum papillosum*	*Sphagnum magellanicum*
Sphagnum fuscum	*Sphagnum pulchrum*	
Sphagnum majus	*Sphagnum tenellum*	
Sphagnum rubellum		
Non–Sphagnum mosses and lichens		
Dicranales	*Hypnales*	*Bryales*
	Cladonia spp.	*Polytrichales*

* including *E. hermaphroditum*
† including *E. ciliaris*

Supplementary Table 3. List of European peat bog plants. List comprising all taxa that were identified in the 56 European peatlands, and included in the final data set.

Vascular plants

Andromeda polifolia
Betula nana
Betula pubescens
Calluna vulgaris
Carex dioica
Carex echinata
Carex lasiocarpa
Carex nigra
Carex limosa
Carex pauciflora
Carex rostrata
Chamaedaphne calyculata
Drosera anglica
Drosera intermedia
Drosera rotundifolia
*Empetrum nigrum (*incl. *E. hermaphroditum)*
*Erica tetralix (*incl. *E. ciliaris)*
Eriophorum angustifolium
Eriophorum vaginatum
Galium palustre
Ledum palustre
Menyanthes trifoliata
Molinea caerulea
Narthecium ossifragum
Picea abies
Pinus mugo
Pinus sylvestris
Potentilla erecta
Rhynchospora alba
Rubus chamaemorus
Scheuchzeria palustris
Trichophorum cespitosum
Vaccinium oxycoccus
Vaccinium uliginosum
Vaccinium microcarpon
Vaccinium myrtillus
Vaccinium vitis-idaea

Bryophytes

Sphagnum angustifolium
Sphagnum austinii
Sphagnum balticum
Sphagnum capillifolium
Sphagnum compactum
Sphagnum cuspidatum
Sphagnum fallax
Sphagnum flexuosum
Sphagnum fuscum
Sphagnum lindbergia
Sphagnum magellanicum
Sphagnum majus
Sphagnum papillosum
Sphagnum pulchrum
Sphagnum rubellum
Sphagnum rusowii
Sphagnum tenellum

Bryales
Funariales
Hypnales
Polytrichales

Cladonia spp.

Plant species omissions. To limit the influence of rare species on our analyses, species occurring in five or fewer peatlands were not included in the dataset. For the vascular plant community, these species were: *Betula pubescens*, *Calamagrostis laponica, Carex dioica, C. echinata, C. lasiocarpa, C. nigra*, *C. limosa*, *C. rostrata, Chamaedaphne calyculata, Galium palustre, Menyanthes trifoliata, Melampyrum sylvestris, Myrica gale, Picea abies* (sapling)*, Pinus mugo* (sapling)*, Pinus sylsvestris, Potentilla erecta*, *Vaccinium myrtillus,* and *Vaccinium vitis-idaea.* For the brypophytes the omitted species were: *Sphagnum compactum, S. lindbergia,* and *S. rusowii. Empetrum hermaphroditum* and *E. nigrum*, and *Erica ciliaris* and *E. tetralix* were grouped at genus level. Non-*Sphagnum* mosses and Cladonia species were grouped at the family level.

Supplementary Table 4. Peat moss trait values**.** Trait values (means) for *Sphagnum* spp. measured for this paper, additioned with data from Bouman (Bouman, A. C. *De Nederlandse Veenmossen*. KNNV uitgeverij (2002)). C = tissue carbon ontent (mg g^{-1}), N = tissue nitrogen content (mg g^{-1}), P = tissue phosphorus content (mg g^{-1}), St.w = stem width (mm), l.h.c. = length hyaline cells (µm), w.h.c. = width hyaline cells (µm), l.s.l. = length stem leaves (mm), w.s.l. = width stem leaves.

	**C**	**N**	**P**	**St.w**	**l.h.c**	**w.h.c**	**l.s.l.**	**w.s.l**
S. angustifolium	456.0	6.60	0.63	0.73	90	12	1.3	0.4
S. balticum	468.1	7.36	0.28	0.50	100	12	0.9	0.7
S. capillifolium	462.3	8.50	0.39	0.52	90	14	1.4	0.7
S. cuspidatum	460.5	10.21	0.38	0.58	130	11	1.3	0.8
S. fallax	462.§	11.28	0.54	0.61	95	16	0.9	0.8
S. fuscum	477.2	6.04	0.43	0.50	135	21	1.2	0.6
S. majus	462.5	8.68	0.40	0.73	125	13	1.2	0.8
S. papillosum	456.9	8.31	0.44	0.70	140	35	1.6	1.1
S. pulchrum	NA	NA	NA	0.63	90	12	1.6	0.7
S. rubellum	467.9	6.67	0.43	0.50	80	12	1.2	0.7
S. tenellum	465.7	9.95	0.46	0.40	75	16	1.0	0.6
